# Quality of Life (QoL) of Children and Adolescents Participating in a Precision Medicine Trial for High-Risk Childhood Cancer

**DOI:** 10.3390/cancers14215310

**Published:** 2022-10-28

**Authors:** Kate Hetherington, Claire E. Wakefield, Kavitha P. K. Kunalan, Mark W. Donoghoe, Brittany C. McGill, Joanna E. Fardell, Rebecca Daly, Rebecca J. Deyell, David S. Ziegler

**Affiliations:** 1 Discipline of Paediatrics, School of Clinical Medicine, UNSW Medicine and Health, UNSW Sydney, Kensington, NSW 2052, Australia; 2 Behavioural Sciences Unit, Kids Cancer Centre, Sydney Children’s Hospital, Randwick, NSW 2031, Australia; 3 Stats Central, Mark Wainwright Analytical Centre, UNSW Sydney, Sydney, NSW 2052, Australia; 4 Western Sydney Youth Cancer Service, Westmead Hospital, Westmead, NSW 2145, Australia; 5 British Columbia Children’s Hospital, Vancouver, BC V6H 3N1, Canada; 6 Children’s Cancer Institute, UNSW Sydney, Sydney, NSW 2052, Australia; 7 Kids Cancer Centre, Sydney Children’s Hospital, Randwick, NSW 2031, Australia

**Keywords:** childhood cancer, quality of life, precision medicine, EQ-5D-Y, parent-proxy

## Abstract

**Simple Summary:**

We assessed quality of life in patients participating in Australia’s national clinical trial of precision medicine for children with high-risk cancer. Quality of life measures aim to capture an individuals’ perceptions of physical, psychological, and social aspects of health. Knowledge of patient quality of life can help clinicians and parents make decisions in a setting where survival time must be weighed against patients’ experiences of illness and treatment. We found that most patients experienced compromised quality of life at the time of trial enrolment, typically in multiple domains. In patients whom we were able to follow-up after receipt of trial sequencing results, this did not change. We found an association between the outcomes of trial testing and patient quality of life which warrants unpacking in future research. Integrating collection of patient quality of life data into clinical processes would provide a more complete picture.

**Abstract:**

Precision medicine is changing the treatment of childhood cancer globally, however little is known about quality of life (QoL) in children and adolescents participating in precision medicine trials. We examined QoL among patients enrolled in PRISM, the Zero Childhood Cancer Program’s precision medicine trial for high-risk childhood cancer. We assessed patient QoL via self-report (aged 12–17 years) and parent-proxy (aged 4–17 years) completion of the EQ-5D-Y. We analysed data using descriptive statistics and regression models. Patients (*n* = 23) and parents (*n* = 136) provided data after trial enrolment and following receipt of trial results and treatment recommendations (*n* = 8 patients, *n* = 84 parents). At enrolment, most patients were experiencing at least some difficulty across more than one QoL domain (81% patient self-report, 83% parent report). We did not find strong evidence of a change in QoL between timepoints, or of demographic or disease factors that predicted parent-reported patient QoL (EQ-VAS) at enrolment. There was strong evidence that receiving a treatment recommendation but not a change in cancer therapy was associated with poorer parent-reported patient QoL (EQ-VAS; Mdiff = −22.5, 95% CI: −36.5 to −8.5, *p* = 0.006). Future research needs to better understand the relationship between treatment decisions and QoL and would benefit from integrating assessment of QoL into routine clinical care.

## 1. Introduction

Precision medicine uses next-generation sequencing techniques to understand the genetic and biochemical profiles of a patient’s disease to tailor treatments [1,2]. Precision medicine is changing our understanding of childhood cancer and is likely to become standard of care in high-income countries in coming years [1,2]. Precision medicine has the potential to identify new treatment options for children with high-risk/poor prognosis malignancies and is playing an increasing role in the diagnosis and treatment of lower-risk cancers. Despite expanding access to and integration of precision medicine in clinical care, along with its promise of improved clinical outcomes [1], we know little about its impact on patient quality of life (QoL). Based on what we know about QoL in children undergoing standard treatment for cancer [3,4], it is likely that both the precision medicine process and resulting treatment with novel therapies have consequences for patient QoL. 

Patient-reported outcomes, including QoL, are widely recognised as an essential component of the comprehensive assessment of the impact of cancer therapies [5]. QoL is a multidimensional construct, capturing individuals’ perceptions of physical, psychological, and social aspects of health [6]. Knowledge of QoL facilitates a more holistic understanding of patients’ and families’ experience of illness and treatment and can play an important role in clinical decision-making [7,8,9,10]. Previous research has examined the impact of childhood cancer on patient QoL, identifying associations with disease, treatment, and demographic factors [3,4]. While findings vary, QoL has been found to be poorer in patients with certain cancers [3,4], at diagnosis (compared with other stages of disease/treatment) [4,11], and when patients have relapsed or have a poor prognosis [4,12]. Active treatment (compared to having completed treatment) [11,13] and higher treatment intensity (compared with less intense treatment) [12,14,15] have also been found to be associated with poorer QoL, with the emotional and physical side effects of therapy both thought to contribute [9,11,16,17,18,19,20,21]. Very few studies have examined how receiving a targeted or less well-established treatment impacts QoL [22,23]. While emerging data suggests that treatment with an efficacious targeted therapy of shorter duration than standard treatment is likely to benefit QoL [23], there is also potential for less well understood/experimental treatments to negatively impact quality of life (e.g., due to toxicity) [24]. The impact on QoL of participating in a precision medicine trial, when there is uncertainty and heterogeneity surrounding the process and the outcome of testing, remains unknown. 

In addition to the likely physical impact on patient QoL of novel therapeutic agents, several aspects of the precision medicine process may have consequences for QoL. The precision medicine process differs from standard treatment with regard to the potential need for additional and/or larger tissue biopsies [25], the time it can take for analysis to be completed and treatment recommendations generated [25], and the possibility that testing may not produce actionable results/identify any additional treatment options [1,26]. Procedures such as obtaining more and/or larger tissue biopsies may be particularly challenging for children and add to the overall treatment burden experienced [22]. Once sequencing is underway, data from international precision oncology trials indicate return of results takes a median of between 13 and 61 days [2]. Parents of children participating in precision medicine trials for high-risk cancer have identified timing pressures as a source of stress [27]. In the high-risk/poor prognosis setting, patients may be deteriorating quickly, treatment options can be limited and patient health status (i.e., the need for patient’s to be well enough) part of the criteria for inclusion in early phase clinical trials [28]. Adding to this, treatment options identified through precision medicine can have a limited evidence base [4,23,29,30,31] and in some cases be difficult to access [25]. Emerging data suggest that difficulties accessing therapeutic agents are challenging for both parents [27] and clinicians [29,32] and can contribute to the decision not to implement a targeted therapy [33]. Alongside these challenges is the possibility that engaging in the precision medicine process, even if ultimately unsuccessful, may benefit patient QoL by giving a patient and their family hope and reassurance that they have explored all potential treatment possibilities [29,34,35]. Identifying aspects of the precision medicine process which impact patient QoL will inform tailored psychosocial support for patients and families. 

Knowledge of how treatment impacts patient QoL can also help guide clinical decision making [7,8,9,36,37,38], particularly when prognosis is poor and treatment options are limited [30]. Recent experimental research examining clinician and parent decision-making about whether to recommend or participate in genomically guided paediatric cancer care identified patient QoL as a key consideration among both groups [39,40]. Early data from Australia’s national precision medicine program for high-risk cancer indicates that while a novel treatment option was identified for 70% of patients who participated [26], only 30% went on to receive a change in therapy. This is consistent with international data which indicates on average 27% of patients (range 3% to 58%) participating in paediatric precision medicine received targeted therapies on the recommendation of the multidisciplinary tumour board [1,2]. The critical decision-making factors in this process are still to be elucidated [2]. Understanding the likely impact of novel therapeutic agents, participation in early phase clinical trials (when available) and the precision medicine process on QoL will make an important contribution to supporting clinician and family decision making in this expanding area. 

Despite the importance of understanding patient QoL, it is currently insufficiently investigated in paediatric clinical trials [41,42,43,44,45]. This is likely due in part to the challenges associated with measuring QoL in paediatric contexts, particularly when coupled with severe illness. While patients are the ideal reporters of their experience, self-reporting may not be feasible when patients are young, ill or cognitively impaired [19,46]. In such contexts parent-proxy reports are a useful and validated approach [19,47], although concordance between parent and patient reporting varies [8,48,49,50,51]. 

To better understand QoL among children and adolescents enrolling in a precision medicine trial for high-risk/poor prognosis cancer we examined patient self-report and parent-proxy reports to answer the following questions:

1. What proportion of patients have difficulty in QoL domains at enrolment in a precision medicine trial for high-risk childhood cancer, and following receipt of trial results?
2. Does patient QoL change from enrolment to following receipt of trial results?
3. What are the predictors of patient QoL at trial enrolment and following receipt of trial results?
4. What is the concordance between parent-proxy and patient self-report QoL?

## 2. Materials and Methods

### 2.1. Study Design

This study reports on a subset of data collected as part of PRISM-Impact, a prospective, mixed-methods study examining families’ and health professionals’ experiences of the PRISM (PRecISion Medicine for Children with Cancer) clinical trial [26] (ethical and governance approval number: HREC/17/HNE/29). PRISM is a national clinical trial for children with high-risk cancer delivered through the ZERO Childhood Cancer Program (Australian/New Zealand Clinical Trials Registry: NCT03336931). PRISM aims to assess the utility and early clinical effects of precision medicine in patients under 21 years of age with high-risk malignancies. PRISM involves germline and tumour whole genome sequencing, RNA sequencing, DNA methylome analysis of patient tumour/cancer samples and generation of in vitro and in vivo personalised tumour models [26]. A national multidisciplinary tumour board (MTB) meets virtually to review each patient’s results, and any potential treatment recommendation identified. Therapy changes are only recommended if age-specific safety data are available and treatment could be accessed through clinical trials, compassionate access, or off-label use [26]. Each treatment recommendation was given a tier from 1 (highest) to 5 (lowest) [26]. Following the MTB meeting, the patient’s treating oncologist shares the PRISM results and any treatment recommendations with the family, who decide together whether to initiate any changes in the child’s treatment. At the time of this study, it took an average of 10.4 weeks (SD 2.8; range 5–20) from patient enrolment in PRISM to MTB report generation, and an average of 13.6 weeks (SD 5.6; range 5–32) from patient enrolment to sharing of the resulting report with the family.

### 2.2. Participants

Patients were eligible for PRISM if they were aged ≤21 years of age and were diagnosed with a malignancy with an expected likelihood of survival of <30% and a predicted life expectancy >6 weeks. Enrolment of high-risk patients was consecutive through paediatric hospitals around Australia. All patients aged 12–17 years enrolled in PRISM and all parents whose child was enrolled in PRISM were invited to participate in PRISM-Impact. Self-report data were not collected from patients age <12 to ensure that patients had sufficient reading levels to complete the questionnaires. While the PRISM clinical trial recruited patients aged from 18 to 21 years the consent procedures and questionnaires differed for patients age 18+ and will be reported separately. Parent participation was not dependent on their child’s participation. Patients and parents were eligible to participate in PRISM-Impact if they had sufficient English language skills to consent and participate and were not experiencing any significant mental health problems. Patients were excluded if their doctor or parent deemed them too unwell to participate (however, their parents were still eligible to participate). 

### 2.3. Procedures

Participants opted into PRISM-Impact through the main study consent form. Two weeks after trial enrolment, we contacted parents who opted in to confirm their and their child’s interest and assess their preferred questionnaire format (online via Qualtrics™ or paper-based). We sent participants the Time 0 (T0) questionnaire shortly after enrolment, followed by the Time 1 (T1) questionnaire once the PRISM study database indicated a patient’s results had been shared with the family. We followed up missing questionnaires with a maximum of three phone calls, after which time we deemed a participant unreachable. We obtained trial data from the PRISM study database (described below) prior to data analysis.

### 2.4. Data Collection and Measures

#### 2.4.1. Demographics

We collected parent and patient demographic information in the T0 questionnaire. Parent demographics included age, gender, highest level of education, employment status, first language, religion, rurality, number of children and household income. Patient demographic data included gender and age.

#### 2.4.2. Clinical Information

We collected clinical information about the patient from the PRISM study database including their initial cancer diagnosis (sarcoma, central nervous system-CNS, leukaemia/lymphoma, neuroblastoma or other), age at diagnosis, and the number of relapses the patient had experienced prior to enrolling in PRISM.

#### 2.4.3. MTB Outcomes

We obtained information about each patient’s PRISM experience from the PRISM study database, including whether their PRISM report included any treatment recommendations (yes or no), and whether a recommended change in therapy was acted on (yes or no).

#### 2.4.4. Patient QoL

We assessed patient QoL at T0 and T1 using the EQ-5D-Y [52] via self-report for patients age 12–17 years and parent-proxy report for patients age 4–17 years. The EQ-5D-Y assesses the extent to which a respondent is experiencing problems (no problems, some problems, a lot of problems) across five dimensions (mobility, self-care, doing usual activities, pain or discomfort, and feeling worried, sad or unhappy). It also includes the EQ visual analogue scale (EQ VAS), which assesses a respondents’ overall health on a scale from 0–100 (where 0 = ‘the worst health you can imagine’ and 100 = ‘the best health you can imagine’). It assesses the same dimensions as the EQ-5D-3L but uses more child friendly language. EQ-5D-Y data can be used to generate health profiles (showing level of difficulty by domain), overall health status (EQ-VAS) and index values (for comparison with country specific values sets when available) [52]. The self-report and proxy versions of the EQ-5D-Y have been found to have moderate to high reliability [53,54,55] and adequate sensitivity to detecting changes over time [56,57]. It has been shown to have good discriminant and convergent validity [53,58], including in a sample of Australian adolescents [59]. The EQ-5D-Y has been used in studies of paediatric health, including chronic illness [53,56,57,59,60,61,62,63,64,65]. 

### 2.5. Data Analysis

We used logistic regression models to examine characteristics associated with parents’ and adolescents’ decision to participate in PRISM-Impact, and characteristics associated with parents maintaining participation at T1. We used descriptive statistics including means and percentages to examine participant demographics, patient QoL at T0 and T1, number of QoL domains affected, and proportion of patients whose reported level of difficulty was the same or differed from T0 to T1 for each QoL domain. We used mixed-effects ordinal regression models (with random effects for participant, nested within family) to estimate the differences over time in the rates of parent-proxy reported problems in each domain of patient QoL, assessing the appropriateness of the proportional odds assumption by comparing these models to similar nominal logistic regression models. We used a similar mixed-effects linear regression model for analysis of change in EQ-VAS overall health rating. We used mixed-effects linear regression models to examine predictors of parent-reported patient overall health (EQ-VAS) separately at T0 and T1. At T0 we included patient age, gender, diagnosis, relapse as variables in the model. At T1 we included parent-proxy overall health (EQ-VAS) at T0, whether the patient received any treatment recommendations from the PRISM trial, and whether the PRISM results led to a change in the patient’s cancer therapy. In these analyses we included a random family effect to account for clustered responses from parents of the same child. Our sample of patient self-report data at T1 (*n* = 8) was too small to allow us to test for change over time or factors associated with QoL. 

We examined concordance between self-report and parent-proxy QoL where we had data on an individual from both sources. We looked at mother-child pairs and father-child pairs separately due to the difficulty in accounting for non-independence within families in a small sample. We examined concordance between each domain of the EQ-5D-Y using the weighted kappa measure of agreement (using quadratic weights) [66] and between overall health (EQ-VAS) using the concordance correlation coefficient [67]. We conducted analyses using SPSS (v26.0) and R (v4.1.2) [68].

## 3. Results

273 families from a possible 420 families opted into the PRISM-Impact study at the time of consenting to the PRISM trial. When we approached these families, at least one family member from 221 families agreed to participate (family response rate of 53%). 

The 221 families included 331 parents who agreed to receive the T0 questionnaire. In total, 136 parents of 109 children aged 4 to 17 years returned the T0 questionnaire (parent T0 participation rate of 41%) and 84 parents of children aged 4 to 17 years returned the T1 questionnaire. At T1, 22 parents of children aged 4 to 17 did not participate due to their child having died (*n* = 18) or being too unwell (*n* = 4). Our data set included reports from two parents for 27 patients aged 4 to 17 years. Analyses examining characteristics associated with parents’ and adolescents’ decision to participate in PRISM-Impact, and characteristics associated with parents maintaining participation at T1 are reported in Supplementary data. 

### 3.1. Participant Demographics (Table 1 and Table 2)

**Figure 1:**
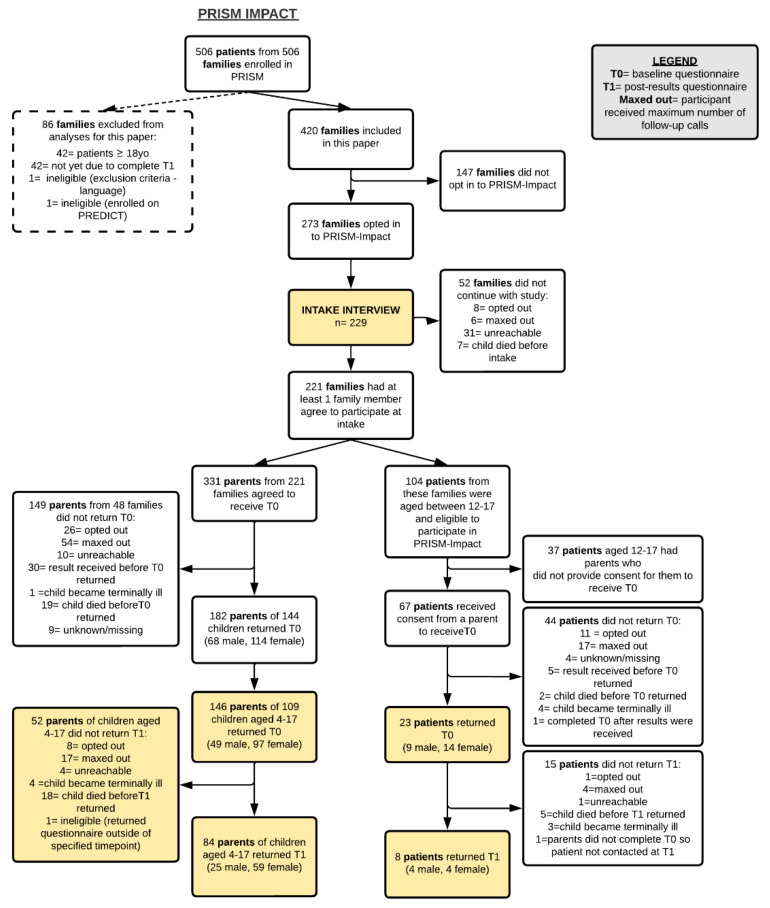
Participant recruitment and flow through PRISM-Impact as of 19 August 2021.

The 221 families included 104 patients who were aged between 12 and 17 years and who were therefore eligible to participate in PRISM-Impact. Parents of 67 eligible patients provided consent for their child to receive the T0 questionnaire (response rate of 64.4%). In total, 23 patients aged 12 to 17 returned the T0 questionnaire (participation rate of 34.3%) and 8 patients aged 12 to 17 returned the T1 questionnaire. At T1, 5 patients aged 12 to 17 had died and 3 indicated they had become too unwell to participate. Figure 1 provides an overview of participant recruitment. 

**Table 1:** Demographics of parents with a child aged 4–17 years at T0 who participated in PRISM-Impact (providing parent-proxy QoL data).

	Parents With a Child Aged 4–17 at T0 (*n* = 136)
**Age, years**	
Mean (SD)	43.6 (6.3)
Median (IQR)	43 (40, 47)
Range	29–67
(missing)	1
**Gender, *n* (%)**	
Female	87 (64%)
Male	49 (36%)
**Highest level of education, *n* (%)**	
High school only	23 (17%)
Post High school (inc. vocational training)	113 (83%)
**Employment, *n* (%)**	
Employed: Full-time	66 (48.8%)
Employed: Part-time/casual	38 (28.2%)
Not employed: Actively seeking work	4 (3%)
Not employed: Not seeking work/retired/student	9 (6.7%)
Not employed: Home duties	18 (13.3%)
(missing)	1
**Cultural or language diversity, *n* (%)**	
First language English, Western/European	102 (77.9%)
First language English, non-Western/European	10 (7.6%)
First language other than English	19 (14.5%)
(missing)	5
**Rurality, *n* (%)**	
Capital city	88 (68.2%)
Other metropolitan centre	10 (7.8%)
Rural/remote area	31 (24%)
(missing)	7
**Marital Status, *n* (%)**	
Currently married or de facto	119 (88%)
Separated/ divorced/ previous de facto	16 (12%)
Widowed	1 (1%)
Never married/ never de facto	0 (0%)
**Household income, *n* (%)**	
Nil income	7 (5.2%)
Less than $29,999	7 (5.2%)
$30,000–$59,000	14 (10.5%)
$60,000–$89,000	28 (20.9%)
$90,000–$120,000	14 (10.5%)
Greater than $120,000	50 (37.3%)
Prefer not to answer	14 (10.5%)
(missing)	2
**Number of other children, *n* (%)**	
0	20 (14.8%)
1	47 (34.8%)
2–3	59 (43.7%)
4+	9 (6.7%)
(missing)	1

SD = standard deviation, *n* = number, QoL = Quality of Life.

**Table 2:** Demographics of patients for whom we have data on QoL at T0.

	Patients Aged 4–17 Years Whose Parents Reported on QoL at T0 (*n* = 109)	Patients Aged 12–17 Years Who Self-Reported on QoL at T0 (*n* = 23)
**Age, years**		
Mean (SD)	11.3 (4.2)	14.8 (1.9)
Median (IQR)	12 (8, 15)	15 (13, 17)
Range	4–17	12–17
**Gender, *n* (%)**		
Female	49 (45%)	14 (61%)
Male	60 (55%)	9 (39%)
**Age of patient at time of diagnosis, years**		
Mean (SD)	10.0 (4.5)	13.0 (2.8)
Median (IQR)	10 (6, 14)	13.5 (11, 15.25)
Range	0–17	7–17
**Diagnosis, *n* (%)**		
Central Nervous System	42 (39%)	5 (22%)
Sarcoma	34 (31%)	12 (52%)
Leukemia/Lymphoma	14 (13%)	4 (17%)
Neuroblastoma	9 (8%)	0 (0%)
Other	10 (9%)	2 (9%)
**Number of relapses for patient prior to PRISM consent, *n* (%)**		
0	46 (42%)	11 (48%)
1	47 (43%)	10 (44%)
≥2	16 (15%)	2 (9%)
**Received cancer treatment while awaiting testing results**		
Yes	87 (79.8%)	20 (87%)
No	22 (20.2%)	3 (13%)
Time (days) from initial cancer diagnosis * to PRISM enrolment		
Median (IQR)	418.5 (11.5, 818)	359 (83, 699)
Range	0–5586	1–4023
Time (days) from cancer event ** resulting in patient being eligible for PRISM and enrolment		
Median (IQR)	6 (1, 16.5)	7 (1, 49)
Range	0–834	0–155

* Some participants have been diagnosed with more than one type of cancer in their lifetime; ** Initial diagnosis, relapse or disease progression; SD = standard deviation; *n* = number; QoL = Quality of Life.

Parents in our sample were mostly mothers (64%) who were an average of 43.6 years old (SD = 6.3, range 29–67). Most had completed some form of post-high school education or training (*n* = 113, 83%), were from an English speaking/Western European cultural background (*n* = 102, 75%), lived in a capital city or other metropolitan centre (*n* = 98, 72%) and were married or living in a de facto relationship (*n* = 119, 88%). Children aged 4 to 17 years for whom we had parent-proxy data were an average of 11.3 years old (SD = 4.2) and more likely to be male (*n* = 60, 55%). Most had been diagnosed with a CNS cancer (*n* = 42, 39%) or a sarcoma (*n* = 34, 31%) and over half had experienced at least one relapse of their cancer (*n* = 63, 58%). They were enrolled in PRISM a median of 6 days (range 0–834) following the cancer event (initial diagnosis, relapse, progression) which resulted in them becoming eligible for the PRISM trial, and a median of 418.5 days (range 0–5586) following their initial cancer diagnosis. Most received cancer treatment while awaiting PRISM testing results (79.8%). Patients aged 12 to 17 years for whom we had self-report data were an average of 14.9 years old (SD = 2.1) and more likely to be female (*n* = 14, 61%). Most had been diagnosed with a sarcoma (*n* = 12, 52%), CNS cancer (*n* = 5, 22%) or leukaemia/lymphoma (*n* = 4, 17%). Roughly half had experienced at least one relapse of their cancer (*n* = 12, 52%). They were enrolled in PRISM a median of 7 days (range 0–155) following the cancer event (initial diagnosis, relapse, progression) which resulted in them becoming eligible for the PRISM trial, and a median of 359 days (range 1–4023) following their initial cancer diagnosis. Most received cancer treatment while awaiting PRISM testing results (87%).

### 3.2. Patient QoL Shortly after Trial Enrolment (Table 3 and Table 4, Figure 2 and Figure 3)

Parent-proxy reported QoL: At T0, most parents reported that their child was experiencing at least some difficulty with: doing their usual activities (*n* = 97, 75%), pain or discomfort (*n* = 89, 69%), and feeling sad, worried or unhappy (*n* = 92, 72%). Approximately half of parents reported their child was experiencing at least some difficulty with mobility (*n* = 66, 51%) and personal care (*n* = 63, 49%). At T0 the number of domains in which parents reported their child was experiencing at least some difficulty ranged from none (9%) to all five (30%). On average, parents rated their child’s overall health as assessed by the EQ-VAS shortly after trial enrolment as 59 (out of 100, SD = 24, range 0–100). 

**Figure 2:**
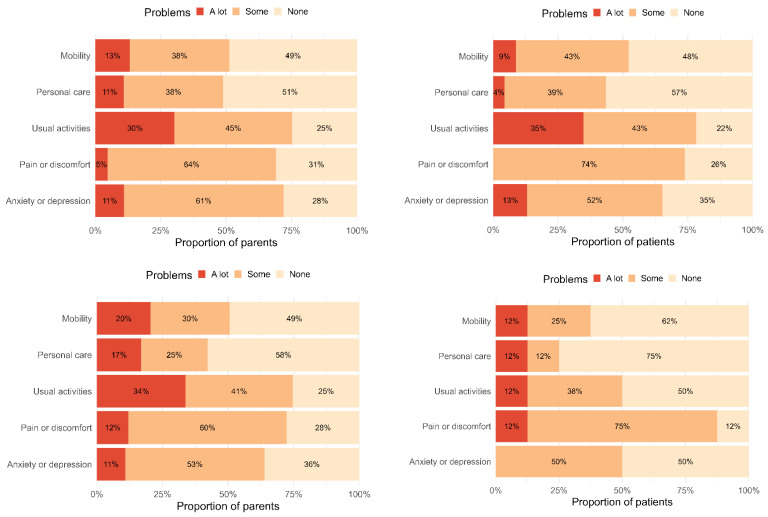
Patient self-report and parent-proxy QoL on EQ-5D-Y domains at T0 and T1.

**Figure 3:**
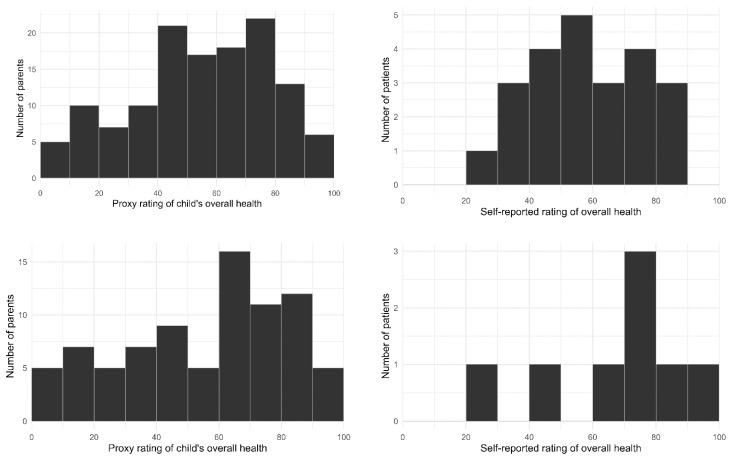
Patient self-report and parent-proxy QoL on EQ-VAS at T0 and T1.

**Table 3:** Patient QoL assessed via parent-proxy and patient self-report on the EQ-5D-Y scale.

	Parent-Proxy Reported QoL for Patients Aged 4–17 Years		Self-Reported QoL for Patients Aged 12–17 Years	
	T0 (*n* = 136) ^1^	T1 (*n* = 84) ^2^	T0 (*n* = 23)	T1 (*n* = 8)
Domain	*n*, Valid %	*n*, Valid %	*n*, Valid %	*n*, Valid %
**Problems with mobility (walking about)**				
None	63 (49%)	4 (49%)	11 (48%)	5 (63%)
Some	49 (38%)	25 (30%)	10 (44%)	2 (25%)
A lot	17 (13%)	17 (21%)	2 (9%)	1 (13%)
**Problems with self-care (looking after him/herself)**				
None	66 (51%)	48 (58%)	13 (57%)	6 (75%)
Some	49 (38%)	21 (25%)	9 (39%)	1 (13%)
A lot	14 (11%)	14 (17%)	1 (4%)	1 (13%)
**Problems doing usual activities**				
None	32 (25%)	21 (25%)	5 (22%)	4 (50%)
Some	58 (45%)	34 (41%)	10 (44%)	3 (38%)
A lot	39 (30%)	28 (34%)	8 (35%)	1 (13%)
**Having pain or discomfort**				
None	40 (31%)	23 (28%)	6 (26%)	1 (13%)
Some	83 (34%)	50 (60%)	17 (74%)	6 (75%)
A lot	6 (5%)	10 (12%)	0 (0%)	1 (13%)
**Feeling worried, sad or unhappy**				
None	36 (28%)	30 (36%)	8 (35%)	4 (50%)
A bit	78 (61%)	44 (53%)	12 (52%)	4 (50%)
Very	14 (11%)	9 (11%)	3 (13%)	0 (0%)

^1^ The domains of Mobility, self-care, usual activities, and pain or discomfort each have 7 missing values. The domain of feeling worried, sad or unhappy has 8 missing values; ^2^ All domains have 1 missing value; n = number; QoL = Quality of Life.

**Table 4:** Number of domains in which patients experience at least some problems (some or a lot).

Number of Domains	Parent-Proxy		Self-Report	
	T0 (N = 128)	T1 (N = 83)	T0 (N = 23)	T1 (N = 8)
	*n* (%)	*n* (%)	*n* (%)	*n* (%)
0	11 (9%)	9 (11%)	0 (0%)	1 (12%)
1	11 (9%)	9 (11%)	4 (17%)	1 (12%)
2	25 (20%)	13 (16%)	4 (17%)	2 (25%)
3	20 (16%)	15 (18%)	4 (17%)	2 (25%)
4	22 (17%)	13 (16%)	7 (30%)	1 (12%)
5	39 (30%)	24 (29%)	4 (17%)	1 (12%)

Patient self-reported QoL: At T0, most patients reported experiencing at least some difficulties with: doing their usual activities (*n* = 18, 78%), pain or discomfort (*n* = 17, 64%) and feeling sad, worried or unhappy (*n* = 15, 65%). Approximately half reported experiencing at least some difficulties with mobility (*n* = 12, 52%) and personal care (*n* = 10, 43%). At T0 the number of domains in which patients reported experiencing at least some difficulty ranged from one (17%) to all five (17%). On average, patients rated their overall health as assessed by the EQ-VAS shortly after enrolment as 61 (out of 100, SD = 17, range 40–89).

### 3.3. Patient QoL following Return of Results and Treatment Recommendations (Table 3 and Table 4, Figure 2 and Figure 3)

Parent-proxy reported QoL: At T1, most parents reported that their child was experiencing at least some difficulty with doing their usual activities (*n* = 62, 75%), pain or discomfort (*n* = 60, 72%), and feeling sad, worried or unhappy (*n* = 53, 64%). Approximately half of parents reported their child was experiencing at least some difficulties with mobility (*n* = 42, 51%), and less than half reported their child was experiencing at least some difficulties with personal care (*n* = 35, 42%). At T1 the number of domains in which parents reported their child was experiencing at least some difficulty ranged from none (11%) to all five (29%). On average, parents rated their child’s overall health (EQ-VAS) following return of results as 59 (out of 100, SD = 27).

Patient self-reported QoL: At T1, most patients reported experiencing at least some difficulties with pain or discomfort (*n* = 7, 88%), half reported at least some difficulties with doing their usual activities (*n* = 4, 50%) or feeling sad, worried or unhappy (*n* = 4, 50%), and less than half reported experiencing at least some difficulties with mobility (*n* = 3, 38%), and self-care (*n* = 2, 25%). At T1 the number of difficulties in which patients reported experiencing at least some difficulty ranged from none (13%) to all five (13%). On average, participating patients rated their overall health (EQ-VAS) following return of results as 70 (out of 100, SD = 21).

### 3.4. Change in QoL from Shortly after Enrolment to Following Receipt of Results (Table 5 and Table 6)

Parent-proxy reported QoL: Among parents who responded at both T0 and T1 (*n* = 84), we found no strong evidence for a change over time in the rates who reported their child was experiencing problems in the EQ-5D-Y domains, although we could not rule out the possibility of small-to-moderate changes in either direction (Table 5). Over half of parents reported their child was experiencing the same level of difficulty within each domain at T1 as at T0 (ranging from 52% to 58%). Among these same parent respondents, we found no evidence for a change in ratings of their child’s overall health (EQ-VAS; *p* = 0.684), which had a fitted mean rating of 57.9 (95% CI: 53.3–62.5) at T0 and 59.1 (95% CI: 53.5–64.7) at T1.

**Table 5:** Change between T0 and T1 in parent-proxy QoL reports on EQ-5D-Y domains *.

Domain	Timepoint	Level of Problems, *n* (%)			OR	(95% CI)	*p*-Value
		None	Some	A lot			
**Mobility**	T0	35 (47%)	26 (35%)	13 (18%)	1.50	(0.74–3.01)	0.260
	T1	34 (46%)	23 (31%)	17 (23%)			
**Self-care**	T0	39 (53%)	27 (36%)	8 (11%)	0.93	(0.45–1.90)	0.840
	T1	43 (58%)	19 (26%)	12 (16%)			
**Usual activities**	T0	19 (26%)	29 (39%)	26 (35%)	1.09	(0.59–2.01)	0.793
	T1	18 (24%)	29 (39%)	27 (36%)			
**Pain/discomfort**	T0	20 (27%)	52 (70%)	2 (3%)	1.55	(0.81–2.97)	0.190
	T1	21 (28%)	44 (59%)	9 (12%)			
**Anxiety/** **depression**	T0	21 (29%)	44 (60%)	8 (11%)	0.72	(0.37–1.41)	0.343
	T1	25 (34%)	41 (41%)	7 (10%)			

* Among parents who responded at both timepoints; QoL = Quality of Life; OR = Odds Ratio; CI = confidence interval.

**Table 6:** Proportion of patients whose QoL increased, decreased, or stayed the same from T0 and T1 by domain *.

Domain			Parent-Proxy			Self-Report		
			T1			T1		
			None	Some	A Lot	None	Some	A Lot
**T0**	**Mobility**	**None**	22 (30%)	11 (15%)	2 (3%)	4 (50%)	1 (12%)	0 (0%)
		**Some**	11 (15%)	9 (12%)	6 (8%)	1 (12%)	1 (12%)	0 (0%0
		**A lot**	1 (1%)	3 (4%)	9 (12%)	0 (0%)	0 (0%)	1 (12%)
	**Self-care**	**None**	29 (39%)	7 (9%)	3 (4%)	6 (75%)	0 (0%)	0 (0%)
		**Some**	14 (19%)	9 (12%)	4 (5%)	0 (0%)	1 (12%)	1 (12%)
		**A lot**	0 (0%)	3 (4%)	5 (7%)	0 (0%)	0 (0%)	0 (0%)
	**Usual activities**	**None**	10 (14%)	6 (8%)	3 (4%0	1 (12%)	2 (25%)	0 (0%)
		**Some**	6 (8%)	14 (19%)	9 (12%)	2 (25%)	1 (12%)	0 (0%)
		**A lot**	2 (3%)	9 (12%)	15 (20%)	1 (12%)	0 (0%)	1 (12%)
	**Pain/** **discomfort**	**None**	7 (9%)	12 (16%)	1 (1%)	1 (12%)	1 (12%)	1 (12%)
		**Some**	14 (19%)	31 (42%)	7 (9%)	0 (0%)	5 (62%)	0 (0%)
		**A lot**	0 (0%)	1 (1%)	1 (1%)	0 (0%)	0 (0%)	0 (0%)
	**Anxiety/** **depression**	**None**	12 (16%)	9 (12%)	0 (0%)	2 (25%)	0 (0%)	0 (0%)
		**Some**	12 (16%)	28 (38%)	4 (5%)	2 (25%)	4 (50%)	0 (0%)
		**A lot**	1 (1%)	4 (5%)	3 (4%)	0 (0%)	0 (0%)	0 (0%)

* Among parents/patients who responded at both timepoints. 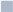 Problem category unchanged at T1; 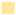 Problem category indicates more difficulties at T1; 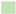 Problem category indicates fewer difficulties at T1.

Patient self-reported QoL: Our patient sample size was too small to enable us to run tests on change over time. Table 3 presents the rates of patients reporting difficulties across the EQ-5D-Y domains and Figure 3 presents patient ratings of overall health (EQ-VAS) at T0 and T1. The mean rating for overall health was 61 (SD = 17) at T0 and 70 (SD = 21) at T1. For patients who responded at both time points, Table 6 presents the proportion who reported experiencing the same/a different level of difficulty for each domain at T0 and T1. The proportion of patients who reported experiencing the same level of difficulty within each domain at T1 as at T0 varied, ranging from 38% (usual activities) to 88% (self-care).

### 3.5. Factors Associated with Parent-Proxy Reported Patient QoL Shortly after Trial Enrolment and Following Receipt of Results (Table 7 and Figure 4)

We found no strong evidence of an association between child demographic (gender, age) and clinical factors (diagnosis, relapse status) and parent-proxy reported patient QoL (EQ-VAS) at T0, but the confidence intervals for these coefficients were wide and included substantial differences in either direction. 

**Figure 4:**
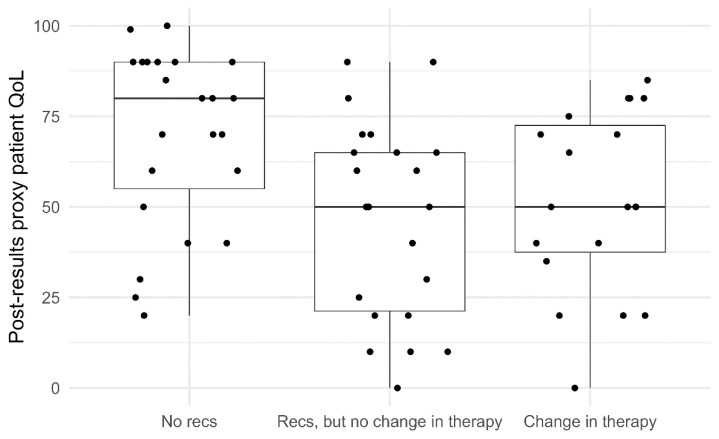
Boxplot distribution of T1 parent-proxy EQ-VAS scores, sorted by recommendations. Dots represent individual data points.

**Table 7:** Estimated associations with parent-proxy EQ VAS at T0 (*n* = 129) * and T1 (*n* = 64) *.

Associations between Characteristics and T0 Parent-Proxy EQ VAS (*n* = 129) *				
Predictor	Response Option	Difference	(95% CI)	*p*-value
Patient’s gender (vs. female)	Male	2.7	(−6.4, 11.9)	0.562
Patient’s diagnosis (vs. CNS)	Sarcoma	−4.3	(−15.5, 6.9)	0.457
	Leukaemia/Lymphoma	−8.0	(−22.2, 6.2)	0.275
	Neuroblastoma	13.2	(−4.6, 31.0)	0.151
	Other	4.2	(−13.3, 21.5)	0.641
Prior relapse (vs. no)	Yes	6.5	(−3.2, 16.3)	0.194
Age	Continuous variable	Smooth relationship		0.509
Associations between MTB Treatment Recommendations and T1 Parent-Proxy EQ VAS (*n* = 64) *				
Predictor		Difference	(95% CI)	*p*-value
Recommendation but no change (vs. no recommendation)		−22.5	(−36.5, −8.5)	0.006
Recommendation and change (vs. no recommendation)		−12.7	(−27.3, 2.0)	0.111

* Among the number of parents who provided proxy VAS scores and had data for all covariates; CNS = Central Nervous System; QoL = Quality of Life; VAS = Visual Analogue Scale.

After controlling for parent-proxy reported patient QoL (EQ-VAS) at T0, we found that receiving a treatment recommendation without a subsequent change in their child’s cancer therapy was associated with poorer parent-reported patient QoL (EQ-VAS), when compared with families who did not receive a treatment recommendation (−22.5, 95% CI: −36.5, −8.5, *p* = 0.006). Receiving a treatment recommendation leading to a change in cancer therapy was also associated with poorer parent-reported patient QoL compared with families who did not receive a treatment recommendation; however, the data was also compatible with a negligible difference in favour of no recommendation (−12.7, 95% CI: −27.3, 2.0, *p* = 0.111).

### 3.6. Concordance between Parent-Proxy Reported Patient QoL and Patient Self-Reported QoL (Table 8)

At T0 there were 22 families in which both the patient and at least one parent reported on the patient’s QoL on the EQ-5D-Y, including 15 families from which one parent had responded, and 7 from which two parents had responded. Given the difficulty accounting for non-independence within families in the small sample, we examined concordance separately for mother-child pairs (*n* = 18) and father-child pairs (*n* = 11). The weighted kappa measure of agreement between mother-child pairs and father-child pairs for each domain ranged in strength from Poor to Excellent. For parent-proxy reported overall patient health ratings on the EQ-VAS at T0 the concordance correlation coefficient was 0.61 (95% CI: 0.26–0.81) for mother-child pairs, and 0.32 (95% CI: −0.15–0.67) for father-child pairs, which are categorised as being of poor strength according to existing guidelines [69]. 

**Table 8:** Parent–child concordance of patient QoL on EQ-5D-Y domains.

Pair Type	Domain	Weighted Kappa	95% CI	Strength of Agreement
**Mother-child**	Mobility	0.94	0.81–1.00	Excellent
	Personal care	0.70	0.39–1.00	Fair-Good
	Usual activities	0.68	0.29–1.00	Fair-Good
	Pain/discomfort	0.00	−0.56–0.56	Poor
	Anxiety/depression	0.13	−0.45–0.70	Poor
**Father-child**	Mobility	0.65	0.23–1.00	Fair-Good
	Personal care	0.37	−0.04–0.77	Poor
	Usual activities	0.25	−0.28–0.79	Poor
	Pain/discomfort	0.42	−0.17–1.00	Fair-Good
	Anxiety/depression	0.26	−0.21–0.72	Poor

QoL = Quality of Life.

## 4. Discussion

This is the first study to report on patient QoL in a sample of young people participating in a precision medicine trial for children and adolescents with high-risk/poor prognosis cancer. Forty percent of patients were enrolled in the precision medicine trial following their initial cancer diagnosis, with the remainder after having experienced at least one relapse. Shortly after trial enrolment, most patients were experiencing at least some problems in more than one QoL domain. Most patients were experiencing at least some difficulty with completing their usual activities, pain or discomfort, and anxiety or depression, and around half were experiencing at least some difficulty with mobility and personal care. Most patients were receiving cancer treatment while awaiting return of their results from the precision medicine trial (an average of 13.6 weeks after patient enrolment). Following receipt of results, parent-proxy data indicated a similar pattern of difficulties as observed shortly after enrolment, and patient self-report data indicated that most were experiencing at least some pain or discomfort, around half were experiencing at least some difficulties with completing usual activities or anxiety or depression, and less than half with mobility and personal care. We did not observe strong evidence of a change in parent-reported patient QoL between timepoints, or of demographic or disease factors that predicted QoL at enrolment. Among patients for whom we had data following receipt of results, having received a treatment recommendation which did not lead to a change in cancer therapy was associated with poorer patient QoL, compared with not receiving a treatment recommendation. 

Our data is consistent with previous studies which have found compromised QoL among children and adolescents undergoing active cancer treatment, including studies focused on the early months of treatment [4,16,18,19,70]. As in previous studies, patients were experiencing difficulties across QoL domains, with some domains more likely to be impacted than others. Our study found completion of usual activities, pain or discomfort and anxiety or depression were the domains in which patients were most likely to be experiencing difficulties. The heterogeneity of measures and participants included in previous research makes it difficult to compare patterns of findings across domains between studies. We identified one study that assessed QoL using the EQ-5D-Y (self-report, Italian translation) in pediatric cancer patients diagnosed with Acute Lymphoblastic Leukaemia (ALL) undergoing chemotherapy maintenance [63]. QoL was poorer in our sample compared with these patients, possibly due to the exclusion of severely unwell patients from the ALL study [63]. The proportion of our sample experiencing problems across domains, and overall, was also higher than that found in other studies that have used the EQ-5D-Y (self-report and/or parent-proxy) in children with other illnesses (including chronic conditions [53], asthma [64], diabetes [60,64], juvenile arthritis [57,64], cystic fibrosis [65], and thalassemia [62]). There were a few exceptions to this pattern, with a greater proportion of young people with Cerebral Palsy reporting problems with mobility [61] than our sample, and patients categorised as acutely ill reporting more or comparable difficulties than our sample across domains other than feeling worried/sad/unhappy [56]. 

Among patients for whom we had data following receipt of results, we found no significant change in QoL over the first few months following trial enrolment. This is consistent with previous studies which have found QoL is most compromised around the time of a child’s diagnosis and remains so in the first few months of treatment [18,19,71], with trajectories after this varying as disease and treatment progress. The absence of a pattern of improvement in patient QoL between timepoints is likely due to our high-risk/poor prognosis sample (i.e., <30% expected survival), variability in where patients were in their disease trajectory at trial enrolment (i.e., following initial diagnosis, relapse, and progression), and the timing of QoL assessment after return of results. While patients received the best available standard treatment while awaiting their precision medicine test results, therapies varied in intensity, length, effectiveness, and burden (with a proportion of patients not receiving treatment between enrolment and receipt of results). Some patients would have been deteriorating medically as a result of their cancer while awaiting testing results, which is likely to have contributed to the pattern of QoL we observed. Participants were invited to complete our second questionnaire shortly after receipt of PRISM results. For some patients this may not have allowed sufficient time for a resulting treatment change to impact QoL prior to assessment. Assessment of patient QoL at T1 will also not have captured the impact of any changes to a patient’s cancer therapy made at a later time, therefore not providing a complete picture of the impact of molecularly informed therapy. 

Our findings focused on QoL after return of results were likely influenced by sample attrition between the two time points. Twenty-two parents who completed T0 had a child who died or had become terminally ill before T1, and 8 patients who self-reported their QoL at T0 died or became terminally ill before T1. While knowledge of QoL among those patients who remained in our sample is useful, the attrition in our sample highlights the need to integrate assessment of patient QoL, and other patient reported outcomes, into routine clinical care in order to gain a more comprehensive assessment of patient QoL and align with recommended psychosocial standards of care [72]. The death of patients prior to their receipt of trial results is also an argument for providing patients with access to tumour sequencing earlier in their disease trajectory in order to meaningfully impact treatment outcomes. 

In contrast with previous research, we did not find strong evidence of an association between demographic (patient age, gender) or disease (diagnosis, relapse status) variables and patient QoL shortly after trial enrolment [3,4,12]. Possibly limiting our sample to patients with a poor prognosis, along with the heterogeneity of our sample in terms of cancer type and point of enrolment (in a patient’s disease trajectory), contributed to the large uncertainty in our results.

We observed an interesting association between the identification/implementation of treatment recommendations and overall patient health (EQ-VAS) among patients for whom we had T1 data. Patients who received a treatment recommendation from PRISM but had no change in cancer therapy had poorer overall EQ-VAS scores than patients who did not receive a treatment recommendation. A similar pattern was evident among patients who received a treatment recommendation and did have a resulting change in cancer therapy, but this association was not statistically significant. Poorer QoL in patients who did not receive a recommended change in therapy is likely to reflect that by the time results were returned, some patients had experienced disease progression (and associated lower QoL) which influenced the decision not to pursue a new treatment option. Disease progression may also have affected patient eligibility for clinical trial opportunities (e.g., decreasing functional status, worsening organ function). This is supported by preliminary analysis of PRISM data, which found that the top three factors which influenced the decision not to adopt a PRISM MTB recommendation were: the patient receiving another therapy, disease progression/death, and inability to access the drug [33]. Challenges with drug access, which have been identified in other studies of paediatric precision medicine [1,2,29], may have contributed to the association we observed between receiving a treatment recommendation with no change in therapy and poorer patient QoL. Parents frequently want to feel like they have done everything possible and have pursued all available options for their child’s treatment [73]. If through their participation in PRISM families became aware of a treatment option but then encountered barriers to access, this could cause psychological distress and potentially negatively impact patient QoL. Related to this, uncertainty is known to be psychologically challenging for families [74,75] and it is possible the precision medicine process increased the uncertainty patients and families faced. The identification of pathogenic and likely pathogenic germline variants (identified in 16.2% of the first 247 PRISM patients [26]), with implications for the patient and their family, may also have impacted patient QoL. Future studies of precision medicine should explore qualitatively which aspects of patient QoL are impacted by precision medicine trials, including both somatically actionable and germline variants, as well as further unpack the association between trial experiences, psychological adjustment and QoL in a larger sample.

Previous studies of the concordance between parent-proxy and patient reports in the EQ-5D-Y scale have had variable findings [56,76,77], with most studies finding higher concordance between observable domains (mobility, self-care, usual activities) than non-observable domains (pain/discomfort, anxiety/depression) with a higher psychological component [56,77,78]. We found this pattern of concordance for mother-child pairs but not father-child pairs, with strength of agreement across QoL domains ranging from Poor to Excellent for mother-child pairs, Poor to Fair-Good for father-child pairs, and a weak correlation between mother-child and father-child reporting on overall health (assessed via the EQ-VAS). We were only able to examine concordance between a subset of our participants, including 18 mother-child pairs and 11 father-child pairs. Discrepancies between parent and child reporting could have resulted from several factors including parents having limited knowledge or awareness of their child’s lived experience, or parents and children having different interpretations of the QoL domains [79].

### Strengths and Limitations

A key strength of our study was its longitudinal design and recruitment of young people as well as parents to report on patient QoL. The recruitment of both participant groups enabled us to include the perspectives of young people themselves, while also including data reflecting the experiences of patients who may have been too young or too unwell to self-report. The use of parent-proxy reporting is a useful and validated method for assessing the experiences of patients with high-risk cancers who are unable to self-report, however, its concordance with patient self-report is variable across studies and can result in over/underestimates. Variability in the strength of concordance across QoL domains was a limitation in our study. Parent-proxy reporting of QoL on the EQ-5D-Y is only validated from age 4 years, meaning we were unable to assess QoL impacts on younger children and infants. Despite the inclusion of both patient and parent data, a limitation of our study was the sample size, particularly the number of patients who participated at both timepoints. This limited our ability to examine change over time and predictors of QoL using self-report data, as well as restricting how many factors we were able to include in our analyses of predictors of parent-proxy reported QoL. In part this was due to attrition of participants at our second timepoint, often as the result of a patient becoming too unwell or dying. As well as limiting our statistical power, attrition may have impacted our results such that patients with very poor QoL may have been more likely to be missing from our second timepoint. When considering our results, it is important to keep in mind that our findings about QoL following receipt of results only reflects those participants who opted into and remained in the study. The lack of a published Australian value set for the EQ-5D-Y limited our ability to calculate standardised index scores for our data. Another potential limitation of our data was its aggregate nature incorporating mixed diagnoses with enrolment at different points in disease trajectories. While this enabled us to examine shared aspects of the precision medicine process, it meant our sample included patients with differing outcomes of sequencing and differing treatments. We were also limited with regard to the clinical predictors of QoL we were able to examine (e.g., histology, prognosis beyond <30% expected likelihood of survival). As precision medicine develops it will be important to develop systems for capturing and responding to QoL and other patient-reported outcome measures at the individual patient level. Our study was also limited by the exclusion of families who were unable to participate in English.

## 5. Conclusions

Our study is the first to examine QoL among patients participating in a precision medicine trial for high-risk childhood cancer. The health status and age of children with high-risk/poor prognosis cancer makes collection of such data inherently challenging, reflected in our sample attrition over time, and our reliance on parent-proxy as well as self-report data. Despite these limitations, our study provides useful insights into QoL among this patient group. Most patients were experiencing compromised QoL across domains, typically in more than one domain. The proportion of patients reporting difficulties in our sample was greater than that reported by patients with other chronic illnesses reported in the literature. Among patients for whom we had data after return of sequencing results, we found no evidence of a change in their QoL over time. Despite the time taken to process patient samples, we observed no deterioration of QoL in most patients for whom we had follow-up data, highlighting the feasibility of the precision medicine process. While we found no demographic or clinical predictors of QoL at enrolment in PRISM, among patients for whom we had data at our second timepoint, receiving a treatment recommendation but no change in therapy was associated with poorer QoL after return of results compared with receiving no treatment recommendation. This may be because by the time results were returned a proportion of patients were deemed too unwell to undergo a change in therapy, suggesting there may be value in sequencing cancers earlier in the trajectory of disease. Our findings highlight the value of identifying how to integrate collection of patient-reported outcomes, including QoL into clinical processes to minimise sample attrition and provide a more complete picture of precision medicine’s impact.

## Data Availability

The data presented in this study are available upon reasonable request from the corresponding author.

## Supplementary Materials

The following supporting information can be downloaded at: https://www.mdpi.com/article/10.3390/cancers14215310/s1.
