# Supplementary material for: Quality of Life (QoL) of Children and Adolescents Participating in a Precision Medicine Trial for High-Risk Childhood Cancer
Source: Cancers (Basel). 2022 Oct 28;14(21):5310. doi: 10.3390/cancers14215310 (PMC9656810; doi:10.3390/cancers14215310)
Supplement: Supplementary file 1 [file cancers-14-05310-s001.zip › cancers-1841182-supplementary.pdf]

Supplementary Data:

Additional sample information:

We examined characteristics associated with parents' and adolescents' decision to participate in PRISM-Impact using logistic regression models (our outcome variable was participation defined as completion of the T0 questionnaire). Covariates included patient age, treating hospital, patient's diagnosis, relapse status. This model found strong evidence that after adjusting for patient age, treating hospital, and diagnosis parents whose child had relapsed had a higher rate of participation than those whose child had not relapsed ($p=<0.001$). For patients, after accounting for other factors in the model there was no strong evidence that participation rates differed by age, treating hospital, cancer type, or relapse status.

We examined characteristics associated with parents maintaining participation in PRISM-Impact at T1 using logistic regression models (our outcome was returning the T1 questionnaire excluding parents who became bereaved, had not yet reached the T1 timepoint or were still being actively followed up, had missing data on tested covariates). Covariates included parent age, parent gender, child's diagnosis (solid or liquid), child's T0 relapse status, whether or not treatment recommendations were included in the child's PRISM report, parent's T0 Emotion Thermometer scores, parent's perception of PRISM-Impact participation as beneficial or burdensome. This model found no strong evidence that maintaining participation at T1 differed according to any of the included demographic or clinical characteristics (all p values=>0.05).
